# Repression of *btuB *gene transcription in *Escherichia coli *by the GadX protein

**DOI:** 10.1186/1471-2180-11-33

**Published:** 2011-02-11

**Authors:** Guang-Sheng Lei, Wan-Jr Syu, Po-Huang Liang, Kin-Fu Chak, Wensi S Hu, Shiau-Ting Hu

**Affiliations:** 1 Institute of Microbiology and Immunology, School of Life Science, National Yang-Ming University, Taipei 11221, Taiwan; 2 Institute of Biological Chemistry, Academia Sinica, Taipei 11529, Taiwan; 3 Institute of Biochemistry, National Yang Ming University, Taipei, 11221, Taiwan; 4 Department of Biotechnology and Laboratory Science in Medicine, National Yang-Ming University, Taipei 11221, Taiwan; 5 Department of Education and Research, Taipei City Hospital, Taipei, Taiwan

## Abstract

**Background:**

BtuB (B twelve uptake) is an outer membrane protein of *Escherichia coli*, it serves as a receptor for cobalamines uptake or bactericidal toxin entry. A decrease in the production of the BtuB protein would cause *E. coli *to become resistant to colicins. The production of BtuB has been shown to be regulated at the post-transcriptional level. The secondary structure switch of 5' untranslated region of *butB *and the intracellular concentration of adenosylcobalamin (Ado-Cbl) would affect the translation efficiency and RNA stability of *btuB*. The transcriptional regulation of *btuB *expression is still unclear.

**Results:**

To determine whether the *btuB *gene is also transcriptionally controlled by trans-acting factors, a genomic library was screened for clones that enable *E. coli *to grow in the presence of colicin E7, and a plasmid carrying *gadX *and *gadY *genes was isolated. The *lacZ *reporter gene assay revealed that these two genes decreased the *btuB *promoter activity by approximately 50%, and the production of the BtuB protein was reduced by approximately 90% in the presence of a plasmid carrying both *gadX *and *gadY *genes in *E. coli *as determined by Western blotting. Results of electrophoretic mobility assay and DNase I footprinting indicated that the GadX protein binds to the 5' untranslated region of the *btuB *gene. Since *gadX *and *gadY *genes are more highly expressed under acidic conditions, the transcriptional level of *btuB *in cells cultured in pH 7.4 or pH 5.5 medium was examined by quantitative real-time PCR to investigate the effect of GadX. The results showed the transcription of *gadX *with 1.4-fold increase but the level of *btuB *was reduced to 57%.

**Conclusions:**

Through biological and biochemical analysis, we have demonstrated the GadX can directly interact with *btuB *promoter and affect the expression of *btuB*. In conclusion, this study provides the first evidence that the expression of *btuB *gene is transcriptionally repressed by the acid responsive genes *gadX *and *gadY*.

## Background

BtuB (B twelve uptake) is a 614 amino acid outer membrane protein of *Escherichia coli*. It is responsible for the uptake of cobalamins [1], such as vitamin B_12 _including cyanocobalamin, hydroxocobalamin, methylcobalamin, and adenosylcobalamin[2]. It also serves as the receptor for bacteriophage BF23 [3]. The synthesis of the BtuB protein in *E. coli *is regulated at the translational level by adenosylcobalamin (Ado-Cbl) which is produced by the BtuR protein (CobA in *Salmonella typhimurium *and CobO in *Pseudomonas denitrificans*) [4-6]. BtuR is an ATP:corrinoid adenosyltransferase and converts cobalamins to Ado-Cbl [4]. In the presence of Ado-Cbl, the stability of the *btuB *mRNA is reduced with a half-life of only 2 - 4 minutes [7]. In addition, Ado-Cbl binds to the leader region (5' untranslated region, 5' UTR) of the *btuB *mRNA and suppresses its translation [8,9]. A 25-nucleotide sequence designated as the B_12_-box located +138 - +162 nucleotides downstream from the transcription initiation site of *btuB *in *E. coli *has been suggested to be the binding site of Ado-Cbl [10]. A B_12_-box is also present in the 5' UTR of both *btuB *and *cbiA *genes of *S. typhimurium *[11]. The *btuB *gene of *S. typhimurium *is highly homologous to that of *E. coli*. The CbiA protein is a cobyrinic acid a, c-diamide synthase using cobyrinic acid as substrate [10,12]. Binding of Ado-Cbl to the 5' UTR of the mRNAs of these genes may interfere with ribosome binding and thus decrease their translation [7-9,13].

It is unknown whether BtuB synthesis is also controlled by regulatory proteins at the transcriptional level. Results of this study suggest that GadX (Glutamic acid decarboxylation) is a transcriptional regulator of *btuB*. GadX has been shown to suppress the expression of *perA *encoded by a plasmid of enteropathogenic *E. coli *[14], but activate *gadX, gadA*, *gadB*, and *gadC *in response to acid stress [15-19]. GadA and GadB are isozymes of glutamate decarboxylases that convert glutamate to γ-aminobutyric acid (GABA) which is then exported by the antiporter protein GadC [20,21]. An intracellular proton is consumed during GABA production [22], but the released GABA, which is less acidic than glutamate, provides local buffering of the extracellular environment.

The expression of *gadX *is activated by the alternative sigma factor RpoS during the stationary phase of growth [15,19,21], but is repressed during the exponential phase by the nucleoid protein H-NS due to its binding to the *gadX *promoter or its destabilizing effect on RpoS [23-25]. However, the acid stress increases the RpoS level and thus induces *gadX *expression even during the exponential phase of growth [26]. GadW, like GadX, belongs to the family of AraC-like regulators. It represses the expression of *gadX *and inhibits the activation of *gadA *and *gadBC *by GadX [15,18,27]. In addition to these trans-acting proteins, the production of GadX is also controlled by *gadY *that is located between *gadX *and *gadW *in an opposite orientation to *gadX *[28,29]. The *gadY *gene has no known protein products. It produces three RNA species of 105, 90, and 59 nucleotides with a common 3' end [28]. The 3' ends of *gadX *and *gadY *RNAs overlap by at least 30 nucleotides and are complementary to each other. Annealing of *gadY *RNA to the 3' end of *gadX *mRNA stabilizes *gadX *mRNA, resulting in an increased production of the GadX protein [28].

BtuB is also involved in the import of colicins such as colicin E7 (ColE7) [30-34]. ColE7 is composed of three domains responsible for the translocation of ColE7 through the OmpF porin, binding of ColE7 to BtuB, and cleavage of DNA [35,36], respectively. The import of ColE7 is dependent on the Tol (tolerance to colicin) system that is composed of TolQ, TolR, TolA, and TolB proteins [35,36]. Deletion or mutation of BtuB, OmpF, or any of the Tol proteins renders *E. coli *resistant to ColE7 [33,37,38]. Based on this information, we used a ColE7 resistance assay in this study to search for transcriptional regulators of *btuB *from a genomic library of *E. coli *strain DH5α and found that *gadX *and *gadY *genes down regulate the expression of *btuB*.

## Results

### Screening of genes conferring *E. coli *resistance to ColE7

**Figure 1:**
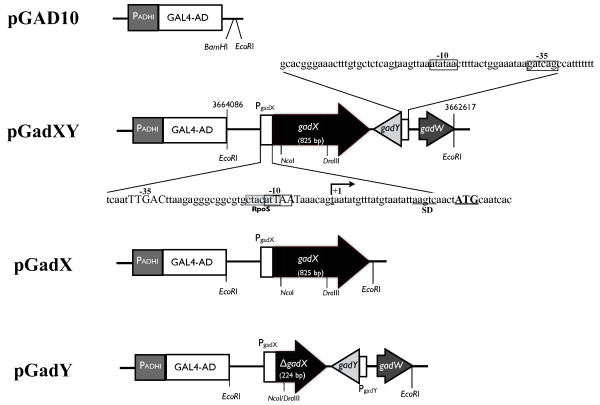
**Structures of pGAD10, pGadXY, pGadX, and pGadY**. pGAD10 was the vector used to clone *gadXY*, *gadX*, and *gadY*. pGadXY has a 1,470-bp fragment containing *gadX*, *gadY*, and a portion of *gadW *of *E. coli *K-12 genomic DNA inserted into the *Eco*RI site of pGAD10. pGadX contains a DNA fragment carrying the 825-bp *gadX *also inserted into the *Eco*RI site of pGAD10. pGadY is derived from pGadXY by deleting the 601-bp *Nco*I-*Dra*III fragment and thus contains a truncated *gadX*, the entire *gadY*, and a portion of *gadW*. Nucleotide sequences of the promoter regions of *gadX *and *gadY *are shown. The orientation of *gadX *is opposite to that of *gadY*. The sigma factor S (RpoS) recognition site and the Shine-Dalgarno (SD) sequence are shown in the 5' end region of *gadX*. P_ADH _is the promoter of GAL4-AD and is not functional in *E. coli*.

To search for genes that can confer *E. coli *resistance to ColE7, plasmids in the genomic library were transformed into the ColE7-sensitive *E. coli *strain DH5α, and the transformants were plated on LB agar plates containing 50 μg/ml of ampicillin and 5.0 ng/ml of His_6_-tagged ColE7/ImE7. Two colonies were seen after incubation at 37°C overnight. The plasmids of each colony were isolated after culturing in 3 ml LB medium containing 50 μg/ml of ampicillin then retransformed into DH5α. The new transformants were plated on agar plates containing 0, 1.3, 2.6, 3.9, or 5.2 ng/ml of His_6_-tagged ColE7/ImE7 to confirm their resistance to ColE7. The insert in the plasmid that conferred DH5α resistance to 5.2 ng/ml His_6_-tagged ColE7/ImE7 was sequenced. A 1,470-bp DNA region on the chromosome at position 3662617 to 3664086 was analyzed that contains both complete *gadX *and *gadY *genes. The plasmid was thus named pGadXY (Figure 1).

To determine whether *gadX *or *gadY *was responsible for ColE7 resistance, pGadX, pGadY, and pGadXY that contain *gadX*, *gadY*, and *gadXY*, respectively, were separately introduced into *E. coli *strain DH5α and then assayed for their ability to confer ColE7 resistance. 1 × 10^5 ^cells containing pGadX, pGadY, or pGadXY were plated on LB agar containing 1.3, 2.6, 3.9, or 5.2 ng/ml of His_6_-tagged ColE7/ImE7. Cells containing the vector pGAD10 were also plated to serve as controls. The percent survival of cells containing pGAD10, pGadXY, pGadX, and pGadY in the presence of 1.3 ng/ml of His_6_-tagged ColE7/ImE7 were 41.7, 95.5, 71.4, and 73.5%, respectively, and 1.5, 63.9, 3.6, and 9.1%, respectively, in the presence of 2.6 ng/ml of His_6_-tagged ColE7/ImE7. Only pGadXY conferred ColE7 resistance to 3.9 and 5.2 ng/ml of His_6_-tagged ColE7/ImE7 with 29.1 and 17.1% survival rates, respectively (Table 1).

**Table 1:** Effects of *gadXY*, *gadX*, and *gadY *on ColE7 resistance

ColE7 conc./Bacteria	pGAD10/DH5α	pGadXY/DH5α	pGadX/DH5α	pGadY/DH5α
1.3 ng/ml	41.7%	95.5%	71.4%	73.5%

2.6 ng/ml	1.5%	63.9%	3.6%	9.1%

3.9 ng/ml	0	29.1%	0	0

5.2 ng/ml	0	17.1%	0	0

### Detection of protein whose expression is affected by *gadXY*

**Figure 2:**
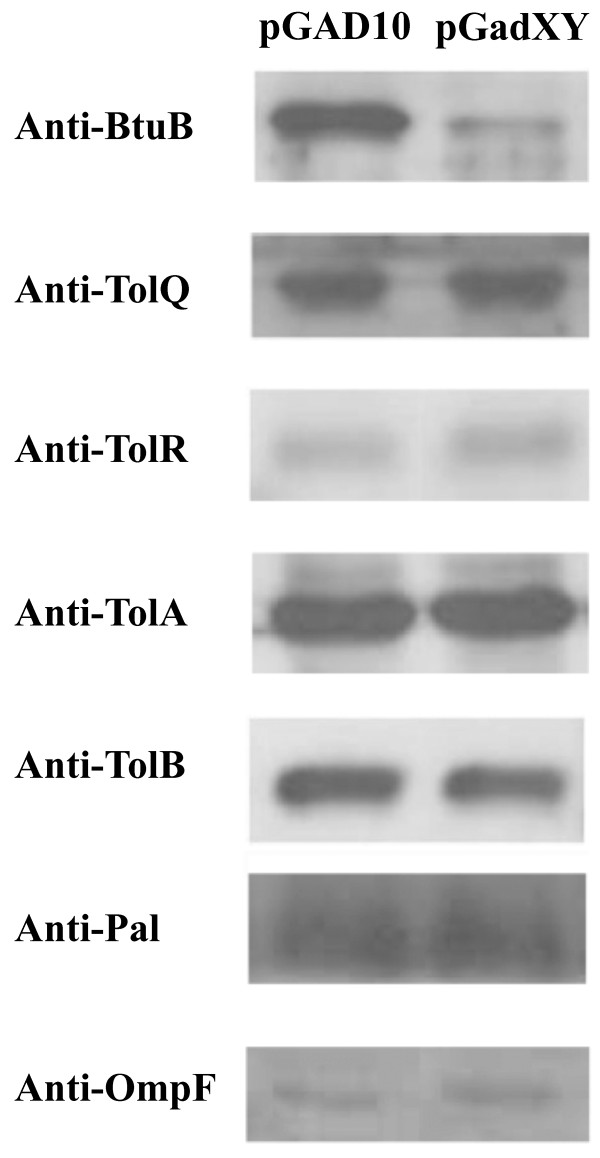
**Effects of *gadXY *on the production of envelope proteins involved in ColE7 uptake**. Cell lysates of *E. coli *DH5α cells harboring pGAD10 or pGadXY were examined by Western blotting using antibodies against envelope proteins BtuB, TolQ, TolR, TolA, TolB, Pal, and OmpF.

To investigate the mechanism by which *gadXY *affects ColE7 resistance, the expression levels of BtuB, TolQ, TolR, TolA, TolB, Pal, and OmpF that are involved in ColE7 import were determined by Western blotting, and BtuB was the only protein found to be affected. Its expression level was reduced by 93% in the presence of *gadXY *(Figure 2) as determined by densitometry.

### Effect of *gadXY *on *btuB *promoter

**Figure 3:**
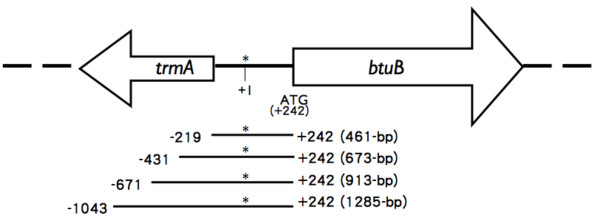
**DNA fragments containing the *btuB *promoter used for *lacZ *fusions**. The *btuB *initiation codon ATG is located at nucleotide position +242. Asterisk indicates the first nucleotide of the *btuB *mRNA. The *trmA *(tRNA methyltransferase) gene is located upstream from *btuB*. It has no known effect on btuB expression.

To determine whether *gadXY *affects the transcription of *btuB*, the β-galactosidase reporter assay was performed. The 461-, 673-, 913-, and 1285-bp DNA fragments (Figure 3) containing the promoter of *btuB *were fused with the *lacZ *coding sequence to generate pCB_461_lacZ, pCB_673_lacZ, pCB_913_lacZ, and pCB_1285_lacZ, respectively. Each of these single copy plasmid together with pGAD10 or pGadXY was transformed into *E. coli *strain DH5α. The transformed cells were grown in LB medium with 50 μg/ml of chloramphenicol and ampicilin to OD_600_~0.8 then assayed for β-galactosidase activity as described by Miller [39]. The β-galactosidase activity of cells containing pGadXY and a pCB derivative with the *btuB *promoter-*lacZ *fusion was divided by that of cells containing the control plasmid pGAD10 and the same pCB derivative to determine the percent decrease in *btuB *promoter activity in the presence of *gadXY*. The *btuB *promoter in the 461-, 673-, 913-, and 1285-bp DNA fragment was found to be decreased by 45.7, 47.1, 54.5, and 56.7%, respectively in the presence of *gadXY*, and was about 6 fold more active in the 1285-bp fragment than in other fragments (Table 2).

**Table 2:** Effect of *gadXY *on *btuB *promoter

Plasmid	**β-galactosidase activity**^**a**^	**% inhibition**^**b**^
(A): pGAD10 + pC-lacZ	0	
(B): pGadXY + pC-lacZ	0	

(A): pGAD10 + pCB_461_lacZ	6.4 ± 0.2	45.7
(B): pGadXY + pCB_461_lacZ	3.5 ± 0.2	

(A): pGAD10 + pCB_673_lacZ	7.2 ± 0.1	47.1
(B): pGadXY + pCB_673_lacZ	3.8 ± 0.1	

(A): pGAD10 + pCB_913_lacZ	4.8 ± 0.2	54.5
(B): pGadXY + pCB_913_lacZ	2.2 ± 0.5	

(A): pGAD10 + pCB_1285_lacZ	37.5 ± 0.7	56.7
(B): pGadXY + pCB_1285_lacZ	16.2 ± 0.5	

^a^Miller unit.

^b^Caculated according to the following equation: 1- [β-galactosidase activity of (B) ÷ β-galactosidase activity of (A)] × 100%.

To investigate the effect of *gadX *or *gadY *alone on the promoter activity of *btuB*, the same experiment was performed using DH5α cells containing pCB_1285_lacZ and pGAD10, pGadXY, pGadX, or pGadY. The β-galactosidase activity of cells containing pCB_1285_lacZ and pGadXY, pGadX, or pGadY was compared to those containing pGAD10 and pCB_1285_lacZ. The results indicated that *btuB *promoter activity was decreased 20.5% by *gadX *and 20.3% by *gadY*, but was decreased 54.4% by *gadXY *(Table 3).

**Table 3:** Effect of *gadX*, *gadY*, and gad*XY *on *btuB *promoter

Plasmids	**β-galactosidase activity**^**a**^	**% inhibition**^**b**^
(A): pGAD10/pC-lacZ	0	

(B): pGAD10/pCB_1285_lacZ	48.8 ± 3.9	

(C): pGadXY/pCB_1285_lacZ	22.3 ± 0.7	54.4

(D): pGadX/pCB_1285_lacZ	38.9 ± 2.6	20.5

(E): pGadY/pCB_1285_lacZ	38.9 ± 2.0	20.3

^a^Miller unit

^b^Calculated according to the following equation: 1- [β-galactosidase activity of (C), (D), or (E) ÷ β-galactosidase activity of (A)] × 100%.

### Binding of GadX to *btuB *promoter

**Figure 4:**
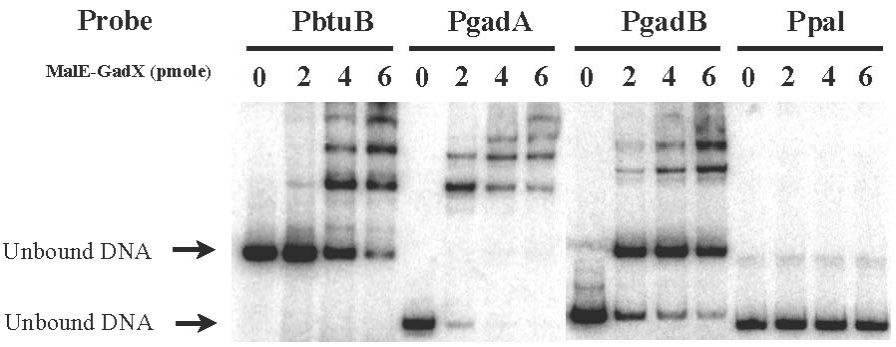
**Binding of GadX to *btuB *promoter**. ^32^P-labeled DNA fragments PbtuB, PgadA, PgadB, and Ppal containing the promoters of *btuB*, *gadA*, *gadB*, and *pal*, respectively, were incubated with GadX fused to the maltose binding protein (MalE-GadX) at 0, 2, 4, or 6 pmoles. The reaction mixtures were electrophoresed in a 5% native polyacrylamide gel. Band shift due to GadX binding was visualized by autoradiography. Arrows indicate bands of DNA probes not bound by GadX.

GadX has been shown to be a DNA binding protein and can bind to the *gadA *or the *gadB *promoter. To determine whether GadX also binds to the *btuB *promoter, the DNA mobility shift assay was performed. Only GadX was assayed because *gadY *does not encode any proteins. The 461-bp DNA fragment containing the *btuB *promoter was labeled with ^32^P and incubated with 2, 4, or 6 pmoles of purified GadX protein (MalE-GadX) that was fused to the maltose binding protein. The DNA fragment containing the promoter of *gadA *or *gadB *was used as the positive control for GadX binding, and the DNA fragment containing the *pal *promoter was used as the negative control. As shown in Figure 4, DNA band shift was observed on *gadA *and *gadB *promoter fragments but not on the negative control. Band shift was also observed on the *btuB *promoter fragment in a dose-dependent manner, indicating that GadX binds to the *btuB *promoter.

### Identification of binding sequence of GadX on *btuB *promoter

**Figure 5:**
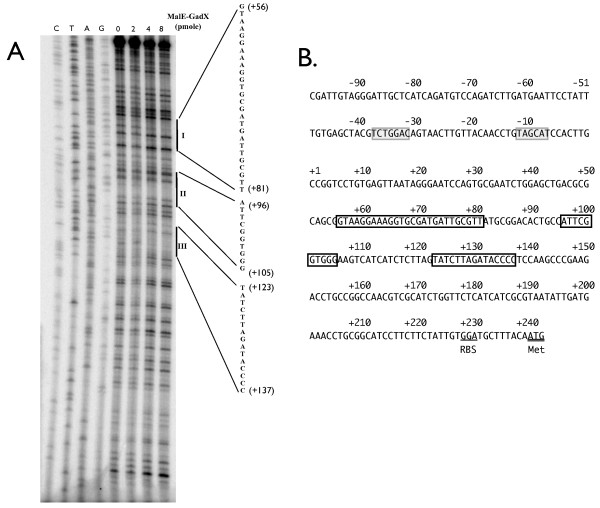
**Binding sequence of GadX on *btuB *promoter**. (A) The 461-bp DNA fragment containing *btuB *promoter was labeled at 5' end with ^32^P, incubated with 0, 16, 24, 32, or 40 pmoles of MalE-GadX, and then subjected to DNase I footprinting. A Sanger's DNA sequencing reaction was also done on the 461-bp fragment to reveal GadX binding sequences. All reactions were electrophoresed in a 6% urea-acrylamide gel, and the DNA bands were detected by autoradiography. The GadX bound regions are indicated with vertical lines, and the binding sequence of GadX are shown. (B) Sequence of the *btuB *promoter region. The boxed sequences are GadX binding sequences determined by the DNase I footprinting. The shaded sequences are -10 and -35 regions of the *btuB *promoter. The initiation codon of *btuB *is underlined.

DNase I footprinting was then performed to determine the binding sequence of GadX on the *btuB *promoter. The 461-bp DNA fragment containing the *btuB *promoter was labeled with ^32^P and incubated with 0, 2, 4, or 8 pmoles of purified MalE-GadX protein and then digested with DNase I. Results shown in Figure 5 revealed three MalE-GadX protein binding sites that included nucleotide positions +56 - +81 (I), +96 - +105 (II) and +123 - +137 (III) on the 5' untranslated region of *btuB*.

### Decreased *btuB *expression under acidic conditions

It is known that *gadX *and *gadY *are more highly expressed under acidic environments in stationary phase [15-19,28]. To determine whether the expression of *btuB *is also repressed in an acidic condition, wild type BW25113 cells were cultured in LB medium pH 7.4 or buffered with 100 mM MES pH5.5. Stationary phase cells grown in different culture media were collected and then assayed for the transcriptional level of *btuB *by quantitative real-time PCR. The cDNA amplification comparison results showed the transcription of *gadX *with 1.4-fold increase but the level of *btuB *was reduced to 57% (Table 4).

**Table 4:** Fold changes of transcripts of *gadX *and *btuB *attribute to different pH medium (pH 5.5/pH 7.4) from early stationary phase.

Gene	**Fold increase**^**a**^
*gadX*	1.43 ± 0.07
*btuB*	0.57 ± 0.13

^a ^Experiments were performed in triplicate and the data are presented as mean values ± SD.

## Discussion

Although it has been suggested that the expression of *btuB *in *E. coli *is also regulated at the transcriptional level, the trans-acting regulators of *btuB *had not been identified [40,41]. In this study, we used the ColE7 resistance assay to search for such regulators and found that both *gadX *and *gadY *genes can repress the production of BtuB rendering *E. coli *DH5α cells resistant to ColE7. Introduction of pGadX, which contains a *gadX *gene, into DH5α cells caused 3.6% of the cells to become resistant to 2.6 ng/ml of ColE7. In a similar experiment, pGadY which contains the *gadY *gene enabled 9.1% of the cells to grow in the presence of the same concentration (2.6 ng/ml) of ColE7 (Table 1). Although *gadY *does not encode any proteins, it had a greater effect on making *E. coli *resistant to ColE7 than *gadX*. This is probably due to the binding of *gadY *RNA derived from pGadY to the *gadX *mRNA produced by the *gadX *gene in the chromosome. This binding stabilizes *gadX *mRNA so that more GadX protein is produced to suppress the production of BtuB, making the cells resistant to ColE7. The greatest effect (63.9% survival in 2.6 ng/ml ColE7) on ColE7 resistance was seen when pGadXY, which contains both *gadX *and *gadY *genes, was introduced into the cells. Since pGadXY is a high copy number plasmid, more *gadX *and *gadY *mRNAs would be produced and thus more GadX protein would be synthesized to suppress BtuB synthesis. However, excess GadX had adverse effects as over expression of GadX with a strong promoter, such as the T5-*lacO *promoter, was found to have toxic effect to *E. coli *[19]. Therefore, expression of *gadX *and *gadY *in this study was driven by their own promoters.

Since GadX is a known transcriptional regulator [14-16,18,19,42], the decrease in BtuB expression is due to its transcriptional repression by GadX. Our data showed that the *btuB *promoter activity was reduced by approximately 50% in the presence of *gadXY *(Table 2), most likely due to binding of GadX to the 5' untranslated region of *btuB *as DNase I footprinting experiment revealed binding of GadX to nucleotides positions +56 - +81 (I), +96 - +105 (II) and +123 - +137 (III) downstream from the *btuB *transcription initiation site (Figure 5). From the sequence alignment of GadX binding sites on *btuB*, *gadA*, and *gadBC *regulatory regions[42], we found that sequence in the region I (the 31 nucleotides) has 62.5% identity (+52-AGCGGTAAGGAAAGGTGCGATGATTGCGTTAT-+82, underlined nucleotides indicate the protected region) with *gadBC *and sequence in the region III (the 26 nucleotides) has 60.7% identity (+106-AAGTCATCATCTCTTAGTATCTTAGATA-+133, underlined nucleotides indicate the protected region) with *gadA *regulatory region. From the footprinting result, the GadX binding sites on 5' untranslated region of *btuB *share only partial homology with the 42 nucleotides consensus sequence which was reported by Tramonti *et. al.*[42]. The sequence analysis also revealed the *btuB *expression was regulated by the binding of GadX on its 5' untranslated region. Binding of transcriptional regulator to the 5' untranslated region to regulate gene expression is also seen in the *glp *regulon of *E. coli*, in which four repressor binding sites are located at -41 to -60, -9 to -28, +12 to -8, and +52 to +33 of the *glpACB *genes [43]. In addition, two IHF binding sites are present downstream from the *glpT *transcriptional start site at positions +15 to +51 and +193 to +227 [44].

In the *btuB *promoter assay experiment, different lengths of DNA fragments containing *btuB *promoter were fused to *lacZ*. The minimum length of DNA fragment with *btuB *promoter activity was 461 bp spanning -219 to + 242 nucleotides relative to the translation initiation site of *btuB*. No significant difference in promoter activity was observed when the 5' end of these fragments was extended to -671. However, a 6 fold (37.5 vs. 6.4 β-galactosidase units, Table 2) increase in promoter activity was detected when the DNA fragment was extended to -1043 with a total length of 1,285 bp as compared to that of the 461-bp fragment. It is very likely that a certain transcription regulator binds to the region between -1043 and -671 and enhances the expression of *btuB*. The β-galactosidase activity in these assays was not very high because the *lacZ *fusions were constructed using the single copy plasmid vector pCC1Bac™ (Epicentre). The purpose of using the single copy number plasmid in this experiment was to mimic the natural state of *btuB *expression in *E. coli*. In fact, the promoter activity of *btuB *is lower than other membrane protein, we have determined the *ompC *promoter activity, under the same test condition the Miller's Units of *lacZ *driven by *ompC *promoter is 8 folds higher than that of *btuB *(data not shown).

Although the results of footprinting and reporter assay revealed that the GadX binding sites on *btuB *5' untranslated region share only partial homology with the GadX binding consensus sequence[42] and showing 50% down regulation in the reporter assay, the expression of *btuB *was indeed controlled by GadX.

Both *gadX *and *gadY *genes belong to a group of genes that are induced by acid stress under stationary growth phase [44]. Our data showed that the expression of *btuB *was indeed reduced when *E. coli *cells were grown to stationary phase in an acidic medium as compared to the same cells grown in neutral medium (Table 4). The reduction in the production of *btuB *in response to acid stress probably represents a physiological regulatory mechanism of bacteria facing environmental challenges such as low pH. Under stress environment, bacteria need to alter their metabolism to adapt to the environmental change. The transportation of cobalamin by BtuB receptor is driven by proton motive force (PMF)[45]. Since the PMF of bacteria is increased at low pH[46], the cobalamin transportation may be enhanced by increased PMF. The higher concentration of cobalamin in cytoplasm will initiate riboswitch mechanism to repress the translation of BtuB receptor, which is in good accord with the repression of *btuB *transcription by the acid-induced GadX for bacteria to decrease the production of BtuB in response to this acidic stress.

## Conclusions

Through biological and biochemical analysis, we have demonstrated the GadX can directly interact with *btuB *promoter and affect the expression of *btuB*. When bacteria were grown to stationary phase in an acidic medium, the increased *gadX *expression would repress the *btuB *transcription to help bacteria to adapt to acidic shock. In conclusion, this study provides the first evidence that the expression of *btuB *gene is transcriptionally repressed by the acid responsive genes *gadX *and *gadY*.

## Methods

### Plasmid constructions

To produce the His_6_-tagged ColE7/Im7 protein complex for the ColE7 resistance assay, pQE30_ColE7-Im7 _was constructed. The *cea7*-*cei7 *genes encoding the colicin E7 and immunity proteins, that form an active ColE7 complex, were amplified from plasmid K317 [47] by PCR using primers F/cea7-*Bam*HI and R/cei-*Pst*I (Table 5). The 1,996-bp PCR product thus generated was inserted between *Bam*HI and *Pst*I sites of pQE30 (Qiagen), fusing the His_6_-tag to the N terminus of ColE7. For searching transcriptional regulators of *btuB*, a genomic library of *E. coli *K-12 strain constructed with the pGAD10 vector (Figure 1) was purchased from Clontech (catalog number XL4001AB) and transformed into *E. coli *strain DH5α. The plasmid pGadXY (Figure 1) was isolated from the library in this study. To investigate the effect of GadX on *btuB *expression, pGadX was constructed as follows. A 1,077-bp DNA fragment containing *gadX *was generated by PCR using pGadXY (Figure 1) as the template and the MATCHMAKER 5' insert screening sequence 5'-TACCACTACAATGGATG-3' (Clontech) and R/gadX-*Pst*I (Table 5) as primers. This 1.1-kb PCR fragment was inserted into pGEM-T_Easy _(Promega) by TA cloning, generating pGEMgadX. The 1.1-kb fragment was then isolated from pGEMgadX by *Eco*RI digestion and inserted into the *Eco*RI site of pGAD10, resulting in pGadX (Figure 1). To investigate the effect of *gadY *on *btuB *expression, pGadY (Figure 1) was constructed by deleting the *Nco*I-*Dra*III fragment containing *gadX *from pGadXY.

**Table 5:** Oligonucleotide primers used in this study

Primer	Sequence 5 '- 3'
F/cea7-*Bam*HI	GGATCCATGAGCGGTGGAGATGGACG
R/cei7-*Pst*I	CTGCAGTCAGCCCTGTTTAAATCC
F/btuB-219-*Xba*I	GGCTCTAGAAAACGGTGCCATCATACTTTG
R/btuB+242-*Hin*dIII	GGCAAGCTTATCATTGTAAAGCATCCACAATAG
F/btuB-767	GTTCACCGTTGCTCGATACC
R/btuB-1087	TCAGATAGATGCCGGTATTACG
F/btuB-431-*Xba*I	GCTCTAGAACGGGATTATTACGC
F/btuB-671-*Xba*I	GCTCTAGATCATCTCTTTCCC
F/btuB-1043-*Xba*I	GCTCTAGACCGCTGCGCGGA
R/lacZ	TTATTTTTGACACCAGACC
F/gadA-176	GATCGCCCGAACAGCAA
R/gadA+77	CGTGAATCGAGTAGTTC
F/gadB-173	AATAACAGCATAAAACA
R/gadB+77	CGTGAATCGAGTAGTTCC
F/pal-*Xba*I	TCTAGAGAGGCGTACAAGTTCTG
R/pal-*Hin*dIII	AAGCTTATCATTTCAATGATTCCTTTAC
F/gadX-*Bam*HI	GGATCCATGCAACCACTACATGG
R/gadX-*Pst*I	CTGCAGCTATAATCTTATTCCTT
F/gadX-393	TATACCGCTGCTTCTGAACG
R/gadX-726	TCGCTCCTGATACTCTGTGG
F/rrsA-483	CGTTACCCGCAGAAGAAGC
R/rrsA-808	GTGGACTACCAGGGTATCTAATCC

The underlined letters indicate restriction sites.

To assay *btuB *promoter activity, DNA fragments (461, 673, 913, and 1,285 bp) containing different portions (Figure 3) of the *btuB *promoter was fused to *lacZ*. These fragments were generated by PCR using primers F/btuB-219-*Xba*I, F/btuB-431-*Xba*I, F/btuB-671-*Xba*I, and F/btuB-1043-*Xba*I paired with the 3' primer R/btuB +242-*Hin*dIII (Table 5). The resulting PCR products were digested with *Xba*I and *Hin*dIII and then inserted into corresponding sites on pKM005 that carries a promoterless *lacZ *gene [48], generating pKMbtuB_461_-lacZ, pKMbtuB_673_-lacZ, pKMbtuB_913_-lacZ, and pKMbtuB_1285_-lacZ. To mimic native expression of *btuB*, these *btuB*-*lacZ *fusions were transferred to the single copy plasmid vector pCC1 (Epicentre). The fragments containing *btuB *promoter and *lacZ *on pKM005 derivatives were amplified with primers F/btuB-219-*Xba*I, F/btuB-431-*Xba*I, F/btuB-671-*Xba*I, and F/btuB-1043-*Xba*I paired with the 3' primer R/lacZ (Table 5), and the resulting 3.3, 3.5, 3.74, and 4.1-kb DNA fragments were separately inserted into pGEM-T_Easy _(Promega) by TA cloning. The 3.3, 3.5, 3.74, and 4.1-kb fragments were then isolated from these pGEM-T_Easy _derivatives by *Not*I digestion and inserted into the *Not*I site of pCC1 vector, generating pCB_461_lacZ, pCB_673_lacZ, pCB_913_lacZ, and pCB_1285_lacZ. The plasmid pC-lacZ that contains a promoterless *lacZ *gene inserted into pCC1 vector was used as a negative control. To produce GadX for DNA binding assay, pMalE-GadX that contains maltose-binding protein fused to GadX (MalE-GadX) was constructed. The 825-bp DNA fragment containing *gadX *was generated by PCR using pGadXY as the template and F/gadX-*Bam*HI and R/gadX-*Pst*I (Table 5) as primers and then ligated between the *Bam*HI and *Pst*I sites of pMAL-C2X (New England Biolab), resulting in pMalE-GadX.

### Production of ColE7

To produce the His_6_-tagged ColE7/ImE7 complex, *E. coli *strain XL1-Blue containing plasmid pQE30_ColE7-Im7 _was cultured in LB medium containing ampicillin (50 μg/ml) and tetracycline (20 μg/ml). When the bacterial growth reached OD_600 _~1.0, IPTG was added to a final concentration of 1 mM. After a 2-hr induction, bacteria were harvested by centrifugation at 6,500 × g for 20 min and then resuspended in HB buffer (20 mM Tris, 150 mM NaCl, 30 mM imidazole, pH8.0). The resuspended bacteria were lysed with a French Pressure Cell (SLM Instruments, Inc. Urbane, IL), and the cell lysate was centrifuged at 48,000 × g for 1 hour. The clarified supernatant was passed through a ProBond™ nickel-nitrilotriacetic acid resin affinity column (Invitrogen, Carlsbad, CA) to purify the His_6_-tagged ColE7/ImE7 according to manufacture's protocol (Invitrogen, Carlsbad, CA).

### Antibody preparation for detection of protein whose expression is affected by *gadXY*

To prepare antibodies against envelope proteins BtuB, TolQ, TolR, TolA, TolB, Pal, and OmpF, the coding region of each protein was fused inframe with His_6_-tag in the plasmid pQE30 (Qiagen), respectively. The His_6_-tagged proteins were then expressed and purified using the same method as described for His_6_-tagged ColE7/ImE7 and sent to the company to prepare polyclonal antibodies. The specificities of these antibodies were confirmed by Western blotting using these antibodies as reported by Pan *et. al*[49].

### DNA binding assay

The electrophoretic mobility shift assay was performed to investigate binding of GadX to the *btuB *promoter. To obtain purified MalE-GadX protein, *E. coli *strain XL-1 Blue containing pMalE-GadX was grown in 100 ml of LB containing ampicillin (50 μg/ml) and 0.2% glucose to OD_600 _~1.0. IPTG was then added to a final concentration of 1 mM. After 2 hr of incubation, the cells were pelleted, resuspended in maltose binding buffer (20 mM Tris-HCl pH 8.0, 200 mM NaCl), and lysed with a French Pressure Cell. The cell lysate was centrifuged at 48,000 × g for 1 hr, and the supernatant was subjected to an amylose resin affinity chromatography (New England BioLabs) to purify the MalE-GadX protein.

To make probes for the DNA binding assay, a 461-bp (Figure 3, -219 - +242) DNA fragment containing the *btuB *promoter was amplified with primers F/btuB-219-*Xba*I and R/btuB+242-*Hin*dIII (Table 5) by PCR. The DNA fragment containing the promoter of *gadA *(-176 - +77, 253 bp) or *gadB *(-173 - +77, 250 bp) was used as the positive control, which were amplified with primer pairs F/gadA-176 and R/gadA+77 and F/gadB-173 and R/gadB+77 (Table 5), respectively, as described by Tramonti *et al. *[19]. The DNA fragment containing the upstream noncoding region of *pal *was used as the negative control, which was amplified with primers F/pal-*Xba*I and R/pal-*Hin*dIII (Table 5). These DNA fragments were end-labeled with [γ-^32^P] ATP by T4 polynucleotide kinase (New England BioLabs). The labeled DNA fragments (6 fmol) were incubated with the MalE-GadX protein at room temperature for 20 min in 10 μl of binding buffer [19]. Samples were then loaded on a 5% nondenaturing polyacrylamide gel in 0.5X TBE buffer and electrophoresed for 35 min at room temperature. The gels were then dried and autoradiographed.

### DNase I footprinting

DNase I footprinting was performed to determine the binding sequence of MalE-GadX on *btuB *promoter as described by Tramonti *et al *[19]. Thirty μl of reaction mixture that contains 5 ng of ^32^P-labeled 461-bp *btuB *promoter fragment, various amounts of the MalE-GadX protein, and reaction buffer (40 mM HEPES pH 8.0, 100 mM potassium chloride, and 10 mM magnesium acetate) was incubated at room temperature for 20 min. At the end of the incubation, 0.5 U DNase I (Roche Biochemicals, Indianapolis, IN) was added to each reaction mixture and then incubated at 37°C for 1 min followed by addition of 3 μl of quench solution (0.1% xylene cyanol, 4% SDS, and 50% glycerol) to stop the DNase I digestion. The partially digested product was passed through a Sephadex G25 spin column (GE Healthcare), and the eluate was subjected to 30 cycles of asymmetric PCR (SequiTherm Excel™II, Epicentre) using 5'-end ^32^P-labeled primer R/btuB+242-*Hin*dIII (Table 5). The PCR-generated products were electrophoresed on a 6% sequencing gel. The gel was then dried and autoradiographed. To determine the binding sequence of GadX, the 461-bp *btuB *DNA probe was sequenced by the Sanger's sequencing method using the 5'-end ^32^P-labeled primer R/btuB+242-*Hin*dIII (Table 5).

### Quantitative Real-Time Polymerase Chain Reaction

Total RNA of wild type *Escherichia coli *strain BW25113 grown under LB (pH 7.4) or LB/MES (LB buffered with 100 mM MES, pH 5.5) to early stationary phase were isolated using a modified hot-phenol extraction method[21]. This was followed by further purification using RNAspin Mini RNA purification kit (GE) to remove contaminating genomic DNA and enhance the quality of RNA. Each cDNA sample was synthesized from 0.1 µg total RNA with specific primers of *rrsA*, *gadX *and *btuB *using RevertAid™ First strand cDNA synthesis kit (Fermentas). Following reverse transcription, specific gene transcription levels were determined by quantitative real-time PCR using the ABI PRISM 7700 Sequence Detection System (Applied Biosystem). Real-time PCR was performed with each specific primer pair using SYBR Green PCR Master mix (MBI). For *rrsA*, primer pair rrsA F and rrsA R was used; for *gadX*, primer pair gadX F and gadX R was used; and for *btuB*, primer pair btub F and btub R was used (Table 5). The rrsA of 16S rRNA was chosen as the normalizing gene. The expression levels of *gadX *and *btuB *of cells grown in medium with different pH and different growth were compared.

## Competing interests

The authors declare that they have no competing interests.

## Authors' contributions

GSL designed and performed most of the experiments, analyzed data and wrote the manuscript. STH designed, supervised all the experiments, analyzed data and wrote the manuscript. KFC provided ColE7 for colicin assay and gave suggestions. PHL provided the antibodies against BtuB, TolQ, TolR, TolA, TolB, Pal, and OmpF for this research. WJS and WSH gave suggestions and analyzed data for this research.

All the authors have read and approved the final manuscript.
